# Activeness and Loyalty Analysis in Event-Based Social Networks

**DOI:** 10.3390/e22010119

**Published:** 2020-01-18

**Authors:** Thanh Trinh, Dingming Wu, Joshua Zhexue Huang, Muhammad Azhar

**Affiliations:** College of Computer Science and Software Engineering, Shenzhen University, Shenzhen 518060, China; dingming@szu.edu.cn (D.W.); zx.huang@szu.edu.cn (J.Z.H.); azhar@szu.edu.cn (M.A.)

**Keywords:** social networks, EBSNs, activeness, loyalty

## Abstract

Event-based social networks (EBSNs) are widely used to create online social groups and organize offline events for users. Activeness and loyalty are crucial characteristics of these online social groups in terms of determining the growth or inactiveness of the social groups in a specific time frame. However, there is less research on these concepts to clarify the existence of groups in event-based social networks. In this paper, we study the problem of group activeness and user loyalty to provide a novel insight into online social networks. First, we analyze the structure of EBSNs and generate features from the crawled datasets. Second, we define the concepts of group activeness and user loyalty based on a series of time windows, and propose a method to measure the group activeness. In this proposed method, we first compute a ratio of a number of events between two consecutive time windows. We then develop an association matrix to assign the activeness label for each group after several consecutive time windows. Similarly, we measure the user loyalty in terms of attended events gathered in time windows and treat loyalty as a contributive feature of the group activeness. Finally, three well-known machine learning techniques are used to verify the activeness label and to generate features for each group. As a consequence, we also find a small group of features that are highly correlated and result in higher accuracy as compared to the whole features.

## 1. Introduction

Event-based social networks (EBSNs) [1], such as Meetup (www.meetup.com) or Douban (www.douban.com), have been rapidly developed as flexible platforms to create many types of online groups that users can join more conveniently than before. The groups often have specific themes, such as writing, cycling, and sports. In order to maintain the groups’ active status, offline activities are often created monthly or even daily, for example, writing events and cycling events, and members are encouraged to attend. Having plentiful options, many users can join different online groups, and they may leave any group after a short period of time. In addition, groups with the same themes and events on similar topics are unavoidable in any kind of social network. Therefore, the shortage of loyal users makes some groups temporarily inactive or even permanently inactive. In contrast, other groups are still stably active and retain loyal users to attend the next events. These realities lead to a new research problem: *activeness and loyalty analysis in event-based social networks*. The research problem is critical for clarifying the existence and growth of these online groups. Thus, a detailed study on this research topic is the need of the current era.

Loyalty is considered as a fundamental concept that represents a relationship between clients and a company [2], as well as a relationship between users and their social groups [3]. Loyal users are considered a crucial factor in keeping their groups active. Therefore, the groups should provide useful services in order to gain new users and retain loyal ones. However, Palla et al. [4] and Jamali et al. [5] found that the tight relationships between users and their groups (small-sized or large-sized) determine the activeness level of the groups. Moreover, the relationships for the activeness of large groups continuously change over time. Hence, the concept of activeness in different social networks is different [6]. For example, active communities in following social networks, such as Instagram or Twitter, can be demonstrated as those who are able to follow many people, but they may be supporters of only one famous person. Similarly, in a citation network, a group of researchers is considered an active community if they refer to a hot research topic. However, the group becomes unstable due to the decrease in their interests in that research topic. The same thing also happens to a group of supporters who are loyal to a celebrity.

In this study, to address the research problem of activeness and loyalty in event-based social networks, we crawled data from the Meetup network in a given time frame. Our objective was to investigate the relationship between user loyalty and group activeness in diversified online groups in EBSNs. We mainly studied the activeness of groups in a series of consecutive time windows since the groups were created in the network. To reveal the characterization of the activeness of groups, we first defined sets of features derived from the crawled data. We then proposed a method to evaluate the activeness level of groups based on a ratio of a number of events between two consecutive time windows. Finally, we developed an association matrix to assign the activeness label for each group after the whole series of consecutive time windows.

Likewise, we measured the loyalty of users in several time windows. For this purpose, we utilized the change in the number of attended events of each user between two consecutive time windows to compute the loyalty of the user towards his/her group. The loyalty of the user was then treated as an essential feature in contributing to group activeness. We used three well-known machine learning techniques to validate the activeness label and the features of each group. We also found a group of correlated features that results in higher accuracy as compared to the features. According to our knowledge, no prior work has studied the problem of activeness and loyalty in EBSNs.

The remainder of this paper is organized as follows. Section 2 briefly reviews related work. Event-based social networks are analyzed, and sets of features are generated in Section 3. The measures of the activeness and loyalty are introduced in Section 4. Section 5 presents the evaluation and analysis of the four real datasets. Conclusions are given in Section 6.

## 2. Related Work

Event-based social networks (EBSNs) had initially been investigated by Liu et al. [1]. Different types of recommendation problems were not only listed, but uniques and interesting characteristics of the networks were also analyzed in their work, such as information flows and locality structural groups. Various recommendation problems were studied in EBSNs [1,7,8,9,10]. However, the problem of activeness and loyalty in EBSNs still needs to be explored.

Detections of active communities and stable links in online social networks have been investigated by many researchers [6,11,12,13,14], and many techniques and methods have been proposed and developed [14,15,16,17,18,19].

The concept of activeness has been studied in several works [6,20,21,22]. In general, activeness presents the state of social processes within a specific time frame; thus, the notion of activeness is understood and bound by a specific period of time. Hence, the status of activeness can be different due to various periods of time: particularly *active*, *stable*, or *inactive*. Detecting active groups in a social network is valuable in understanding the essential characteristics of each group and development of the network. In [6], the authors studied community structures and proposed a framework to detect stable communities in directed social networks, such as Facebook and Twitter. They exploited the mutual links of all connections, and a Markov chain model was used to detect stable communities. Zhang et al. [13] studied a problem of stable link prediction field within a small group of Facebook users evolved in one selected month. They developed a new multiple linear regression model from a single model by using generated multi-variate features of links in that model. Zhang et al. [12] also adopted the multi-variate vector auto regression analysis approach to propose a new stable link detection method that improved the accuracy of stable link detection.

Quintane et al. [20] studied a problem of active social interactions and modeled them as important regularities in different time frames (short-time and long-time) to reveal distinct social processes. They used email datasets obtained from two separate teams in a project to carry out an empirical analysis. They concluded that the time frames were key to discovering the nature of the social processes, and the active process in a short-time period had an impact of social structure on individual, group, and organizational outcomes in a long-time period. In a different work [22], the authors defined a prominent actor as having active participation in one group and attending different groups regularly, and then proposed a new method to identify the actor in time-varying affiliation networks. Moreover, Patil et al. [11] studied a problem of predicting active online groups throughout two different datasets, i.e., an online game network (World of Warcraft) and a large co-authorship network (DBLP). They first examined individuals who had an influence on other users, and then proposed a model to predict the groups of those individuals that can remain stably active, and the groups can shrink over time.

The fundamental concept of loyalty was firstly used in marketing and business areas, where loyalty plays an important central role [2,23]. The notion of loyalty was used as a measure to understand customers’ demands, so that companies can provide better products for their customers and thus gain more profits. Moreover, the development and maintenance of long-term relationships were treated as contributive factors in improving customer loyalty [24]. Gamboa et al. [25] used Facebook users to analyze the customer loyalty of the Zara company, and they created a list of key factors that determined the relationship between users and the company, i.e., trust, satisfaction, and perceived value factors. However, the lack of information in the virtual world caused the user loyalty to be unfaithful [26], and it became one of the major reasons of the inactiveness or collapse of many online groups. In another work, Hamilton et al. [27] studied the concept of loyalty in multiple online groups throughout the Reddit network; the concept was used for both the online community level and the user level. They found that loyal users within active communities have denser social interactions than those within inactive communities.

In our work, we focus on studying the activeness of groups and the loyalty of users. Loyalty is considered a valuable factor that contributes to the activeness of groups. Different from other social networks, event-based social networks do not have direct links between users.

## 3. Data Collection and Feature Generation

In this section, we first introduce the datasets and their structures crawled from the Meetup network. We then present the set of features generated from these obtained datasets.

### 3.1. Meetup Data Collection

**Figure 1:**
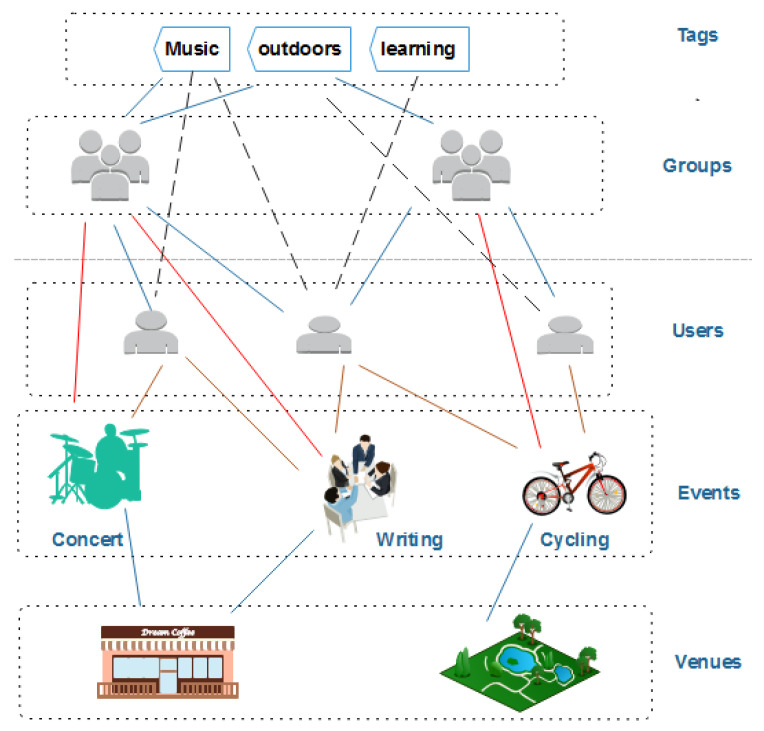
Example of an event-based social network (EBSN).

We crawled data from the Meetup network, which is considered as one of the largest event-based social networks. In March 2019, Meetup had over 40 million users, and more than 10 thousand events were created every day in more than 180 countries through the Meetup network. The structure of EBSNs is demonstrated in Figure 1, which contains five main entities. Each event is created by one specific group and held in a particular venue at a given time. An event can be created by anyone in a group, and the event can be hosted by one or more event organizers. Each event has a list of RSVPs, which presents those who confirm YES or NO to take part in it. Users can join many groups, and each group is created by only one primary organizer.

Four crowded cities in three continents of the world were chosen in this study, i.e., London (LD), Sydney (SN), San Francisco (SF), and New York (NY). The data of these cities were crawled from the Meetup website, containing events only in groups created in 2014 and occurring from the beginning of 2014 to the end of 2016. The information of the users of those groups, who joined at that time, were also obtained. These crawled datasets are summarized in Table 1.

We simply use the group category information gathered from the Meetup network. Table 2 shows 33 predefined categories; the type of each group is assigned in Table 2.

### 3.2. Features

This section presents the features we use in the machine learning models. According to the structure of event-based social networks, we can generate a number of features from three main entities, i.e., group, event, and user entities. For further clarification of the presentation, we divide these features into three categories: group-based, event-based, and user-based features. Table 3 shows a list of generated features.

**Group-based features.** These features are derived from the basic information of each group. The characteristics of these features are discussed in detail as follows.

The CATEGORY feature is obtained from Table 2 for each group, and each group has only one category value. Different to the CATEGORY feature, the N_TOPICS feature denotes a number of topics that the group is interested in. Those topics are selected by the primary group organizer, and they can be manifested by a set of tags, as shown in Figure 1. The number of users in each group is considered an important feature, denoted by N_USERS. This feature N_USERS reflects the growth of groups. In addition to utilize the crawled data, the rating level and creation time of each group are also taken into account. RATING is a score of the average of group reviews, and creation time provides information of the time that a group was created in YEAR, MONTH, DAY_OF_MONTH, and WEEKDAY.

**Event-based features.** These features represent the information of all events created by each group, and reflect the diversity of activities in the group. We create the set of features from this information as follows.

The number of events N_EVENTS in each group is derived as a crucial feature to evaluate the growth of the group. Each event has a set of RSVPs that present those who make a confirmation of whether to attend the event or not. The RSVPs feature presents a sum of all sets of RSVPs in all events created by each group. Furthermore, features Y_RSVPs and N_RSVPs that are obtained from the RSVPs feature present the total number of RSVPs with YES and that with NO, respectively. The AVERAGE_RSVPs and SD_RSVPs features represent the average of all sets of RSVPs and the standard deviation of all sets of RSVPs in the group. Similarly, the AVERAGE_Y_RSVPs, SD_Y_RSVPs, AVERAGE_N_RSVPs, and SD_N_RSVPs features are generated from Y_RSVPs and N_RSVPs. These features also reflect the loyalty of users who take part in events. The AVERAGE_DAY and SD_DAY features present the average of days between two consecutive events and the standard deviation of them for each group. These two features characterize the activity of the group; for example, events are held weekly or monthly. The number of events that have organizers is represented in the N_EVENT_ORGANIZER feature.

**User-based features.** These features reveal the properties of all users in each group. This set also reflects the activities of active users in the group.

The number of organizers, who manage events in the group, is denoted by the N_ORGANIZER feature. The number of users who attend at least one event in each group is defined in the N_ATTENDEES feature, which describes the density of active members in the group. BIO and ADDRESS features are obtained from the users who have biography information and those who have addresses in the crawled datasets, respectively.

## 4. Methodology

In this section, we propose novel methods to measure the activeness of groups and the loyalty of users. To predict the activeness of groups, three well-known machine-learning techniques are used, and results have been shown to validate our methods.

### 4.1. Method to Measure Group Activeness

In the multi-online groups platform like Meetup, one key characteristic that emerges to clarify the active or inactive status of each group is the number of events in a specific period of time. Because of different types of groups, the numbers of events of groups are different in the same time period. For example, some groups have events daily or weekly, while others arrange only one event in a month or even in three months. Hence, the change in the number of events in each group between two consecutive time intervals is adopted to evaluate whether the group is active or not. In other words, the change in the number of events is used to specify three levels of group activeness, i.e., *active*, *stable*, and *inactive*, because this feature strongly reflects the group activeness than any other features. Therefore, we define the concept of group activeness in EBSNs based on the numbers of events that are created in different consecutive time windows. Table 4 lists the notations used in this paper.

To measure activeness, we propose a method that is used to label the activeness level of each group after a set of consecutive time windows. The proposed method consists of two steps. Inspired by calculating the journal impact factor from Clarivate Analytics organization (https://clarivate.com/), first we compute the ratio on the numbers of events in two consecutive time windows and assign each group with an activeness label. After that, we develop an association label matrix to reassign a new label for each group after several consecutive time windows. We do not consider more consecutive windows in deciding group activeness because different groups have different histories. It will complicate the matter if multiple time windows are used to compute the activeness of different groups. The process of this method is expressed in the following details.

**Figure 2:**
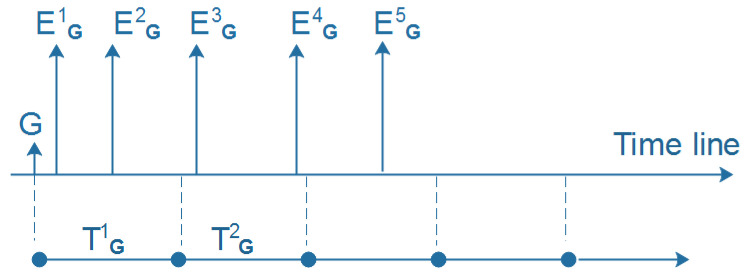
Example of the time frame of group *G*.

**Activeness labels.** Given a group *G* and a given set of consecutive time windows, { $T^{1}$, …, $T^{n}$}, in which each $T^{i}$ has a corresponding number of events $N^{i}$ created by *G*, as illustrated in Figure 2, we first calculate the ratio of events *R* between $T^{i}$ and $T^{i-1}$ as the following equation:

(1) $$R=\frac{N^{i}}{N^{i-1}}$$

where $N^{i}$ and $N^{i-1}$ are the numbers of events of the group *G* in $T^{i}$ and $T^{i-1}$, respectively. Note that, if both $N^{i}$ and $N^{i-1}$ are equal to 0, the value of *R* is assigned to 0. On the other hand, if $N^{i}$> 0 and $N^{i-1}=0$, then the value of *R* is assigned by $N^{i}$.

The label of the group *G* in the period of $T^{i-1,i}$ is then defined as follows:

(2) $$G_{L}=\left\{\begin{array}{cc} A:Active, & \mathrm{if}\;R\;\geq \;1.\\ S:Stable, & \mathrm{if}\;0.5\;\leq \;R\;<\;1.\\ I:Inactive, & \mathrm{if}\;R\;<\;0.5. \end{array}\right.$$

where *R* is the ratio computed in Equation (1), and *L* denotes the labels of the group.

Finally, we develop the following label matrix to assign an activeness label for the group *G* after the whole set of time windows $T^{1,\cdots ,n}$.

$$label\;matrix=\begin{array}{cc} & \begin{array}{c} \;I^{1,\cdots ,n-1}\;S^{1,\cdots ,n-1}\;A^{1,\cdots ,n-1} \end{array}\\ \begin{array}{c} I^{1,\cdots ,n-1}\\ S^{1,\cdots ,n-1}\\ A^{1,\cdots ,n-1} \end{array} & \left[\begin{array}{c} \;I\;I\;S\;\\ \;S\;S\;A\;\\ \;S\;A\;A\; \end{array}\right] \end{array}$$

Specifically, if $S^{1,\ldots n-1}$ is the activeness label of *G* in the period of $T^{1,\cdots ,n-1}$, and $A^{n-1,n}$ is the label in the period of $T^{n-1,n}$. The activeness label of *G* in the whole $T^{1,\cdots ,n}$ is assigned by *A*.

The following example describes the process of assigning the activeness label for groups.

$$A=\begin{array}{cc} & \begin{array}{cccc} \;T^{1} & T^{2} & T^{3} & T^{4} \end{array}\\ \begin{array}{c} G^{1}\\ G^{2}\\ G^{3}\\ G^{4}\\ G^{5} \end{array} & \left[\begin{array}{cccc} 12 & 10 & \;0 & \;0\\ 3 & 1 & \;0 & \;3\\ \;3 & 1 & \;14 & \;10\\ 1 & 2 & \;1 & \;1\\ 13 & 10 & \;14 & \;11 \end{array}\right] \end{array}\to B=\begin{array}{cc} & \begin{array}{c} \;T^{2}/T^{1}\;T^{3}/T^{2}\;T^{4}/T^{3} \end{array}\\ \begin{array}{c} G^{1}\\ G^{2}\\ G^{3}\\ G^{4}\\ G^{5} \end{array} & \left[\begin{array}{c} 0.83\;0.0\;0.0^{*}\\ 0.33\;0\;3^{*}\\ 0.33\;0\;3^{*}\\ 0.33\;14.0\;0.71\\ \;2\;0.5\;1\\ 0.77\;1.4\;0.78 \end{array}\right] \end{array}\to C=\begin{array}{cc} & \begin{array}{c} \;T^{1,2}\;T^{2,3}\;T^{3,4} \end{array}\\ \begin{array}{c} G^{1}\\ G^{2}\\ G^{3}\\ G^{4}\\ G^{5} \end{array} & \left[\begin{array}{c} S\;I\;I\\ I\;I\;A\\ I\;A\;S\\ A\;S\;A\\ S\;A\;S \end{array}\right] \end{array}$$

$$\to D=\begin{array}{cc} & \begin{array}{c} \;T^{1,2,3}\;T^{3,4} \end{array}\\ \begin{array}{c} G^{1}\\ G^{2}\\ G^{3}\\ G^{4}\\ G^{5} \end{array} & \left[\begin{array}{c} I\;I\\ I\;A\\ S\;S\\ A\;A\\ A\;S \end{array}\right] \end{array}\to Activeness\;label=\begin{array}{cc} & \begin{array}{c} \;T^{1,2,3,4} \end{array}\\ \begin{array}{c} G^{1}\\ G^{2}\\ G^{3}\\ G^{4}\\ G^{5} \end{array} & \left[\begin{array}{c} \;I\;\\ \;S\;\\ \;S\;\\ \;A\;\\ \;A\; \end{array}\right] \end{array}$$

**Example of activeness label.** The matrix *A* describes the numbers of events of five groups ($G^{1}$, …, $G^{5}$) in four consecutive time windows. First, the ratios of the numbers of events between two consecutive time windows for each group are calculated as shown in the matrix *B*. Note that the values in the matrix *B* with a marked * are also assigned based on Equation (1). Then, the matrix *C* illustrates the activeness label of each group in the two consecutive time windows, the labels are assigned based on Equation (2). To label these groups in the period of the first three time windows, i.e., $T^{1}$, $T^{2}$, and $T^{3}$, we select the first two columns, i.e., $T^{1,2}$ and $T^{2,3}$, in the matrix *C*. After that, we use the *label matrix* to assign a label to each corresponding group for the period of $T^{1,2,3}$. The results of the five groups for the period of $T^{1,2,3}$ are shown in the first column in the matrix *D*, and the second column $T^{3,4}$ in *D* is still the activeness label obtained from the matrix *C*. Finally, we use the *label matrix* for the matrix *D* to achieve the activeness label for the five groups after the period of $T^{1,2,3,4}$, as illustrated in the *activeness label* matrix.

### 4.2. Method to Measure User Loyalty

Similarly, we use the time windows to define the loyalty of users towards their group. The concept of user loyalty is defined based on participation, which is considered as a strong view for clarifying loyal users. To measure the loyalty of a user *u* in his/her group *G*, we first obtain attended events by user *u* in one $T^{i}$. We then calculate $P^{i}$, which is the ratio of attended events of *u* to all events of *G* created in $T^{i}$. Finally, we measure the loyalty of user *u* after several consecutive time windows as follows.

(3) $$\lambda =\frac{\sum_{i=1}^{n}P^{i}}{n}$$

where *n* is the number of consecutive time windows. The value of λ is from 0 to 1. If the value is close to 1, the user *u* is loyal to the group *G*. Otherwise, if the value is near to 0, the user *u* is the disloyal user.

We use a given threshold τ to differentiate loyal users and disloyal users within *n* consecutive time windows. If λ⩾τ, which means *u* is considered the loyal user; for example, *u* is the loyal user of *G* within $T^{1,2}$. The number of loyal users of *G* is denoted by the LOYAL_USERS feature.

### 4.3. Prediction Techniques

As discussed above, one group has a set of generated features and a corresponding activeness label. The set of these features consists of user-based features, group-based features, event-based features, and the LOYAL_USERS feature. To evaluate the set of generated features and the activeness label for each group, we adopt supervised classifiers. The random forest (RF) method [28,29] can predict high accuracy even with a set of weak features. The decision tree method [30] can select a group of strong features to construct a prediction tree. Moreover, these methods are nonparametric ones. The support vector machine (SVM) [31] is another well-known method that is very useful for a group of features that are highly correlated. Therefore, we selected these three methods to evaluate the activeness label of each group by using the set of features of the group. The process of evaluation is described in Section 5.4.

## 5. Evaluation and Analysis

The datasets that we used in our experiments have been described in Section 3. They contain groups that were created in the year 2014, and all event datasets of each group that were created by the end of the year 2016 were obtained. All users of each group were also selected in that time.

**Figure 3:**
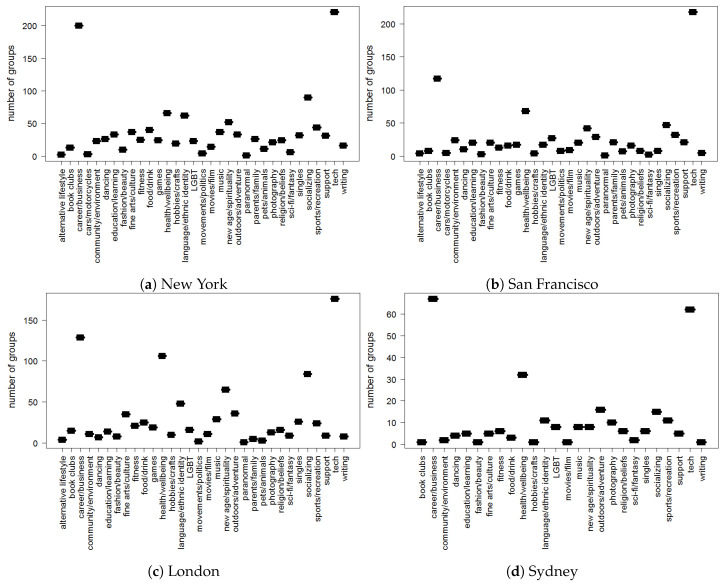
Category type distribution in groups created in four cities in the year 2014.

Figure 3 represents the category type distribution in the online groups created in the four cities in the year 2014. We can observe that a majority of groups belongs to the *tech* and *career/business* category type.

### 5.1. Time Window

**Figure 4:**
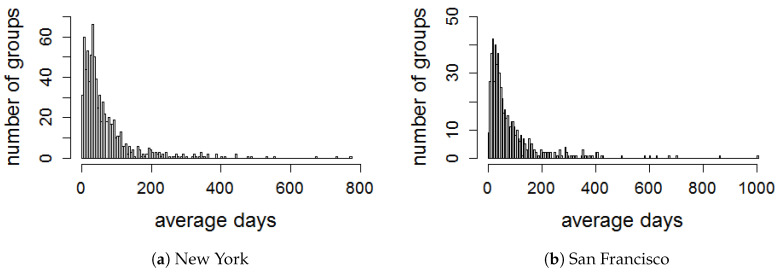

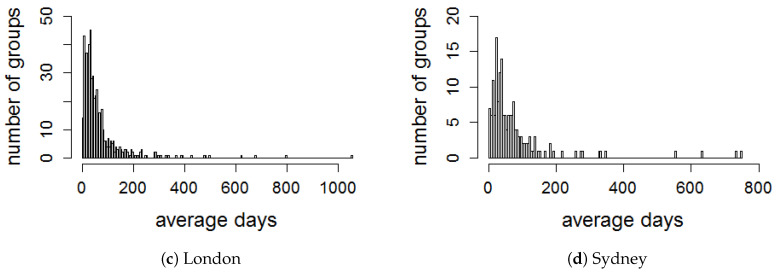
Distribution of average days between two consecutive events in each group.

We evaluate the activeness of groups by dividing a time period of each group in several time windows from its creation date. To select a sliding time window *T* in the lifetime, all events that have been created by each group are obtained in order to compute the average time between two consecutive events in the group. We plot the distribution of average days between two consecutive events created in each group for all datasets, as illustrated in Figure 4. It is observed that the majority of the average time periods are less than 90 days. Therefore, we set *T* with the interval of 90 days to evaluate the activeness of all groups in the four cities. Thus, we create a series of eight consecutive Ts for all groups in all datasets, the series is equivalent to the period of 2 years.

**Figure 5:**
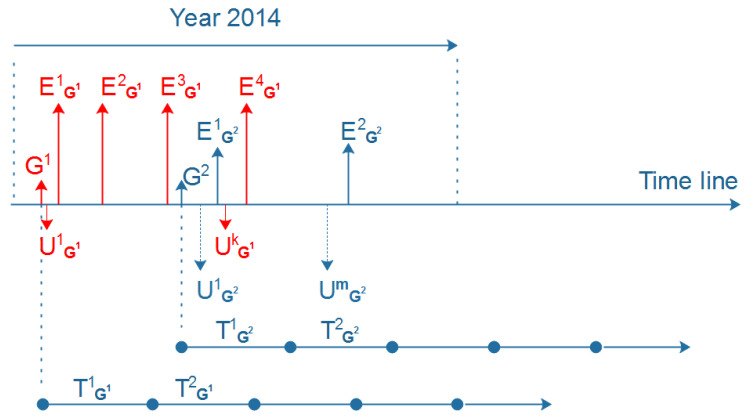
Example of the time window *T* in $G^{1}$ and $G^{2}$.

Figure 5 demonstrates the time windows *T* of group $G^{1}$ and group $G^{2}$. The creation time of these two groups is different; therefore, the starting point and ending point of each *T* for each group are different. Each group has a number of events in each *T*.

### 5.2. Group Activeness Label

For each group *G* in all datasets, we created a corresponding series of 8 time windows, i.e., $T_{G}^{1,\cdots ,8}$. For each $T_{G}^{i}$, we collect all events created in $T_{G}^{i}$ to assign the activeness label for the group. However, we only evaluate groups that create at least one event in the first time window $T^{1}$. If a group does not have any event in $T^{1}$, the group will be removed in the process of evaluation. Otherwise, the group will be taken into the evaluation of activeness.

**Figure 6:**
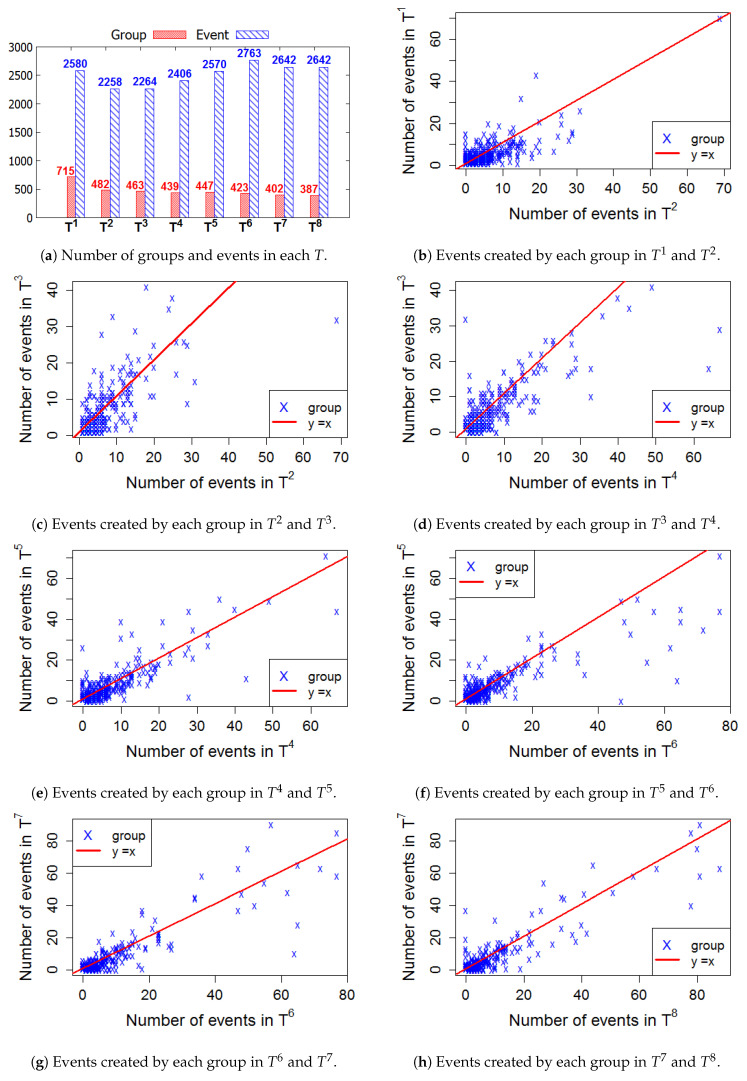
New York—The numbers of groups and events in 8 time windows.

**Figure 7:**
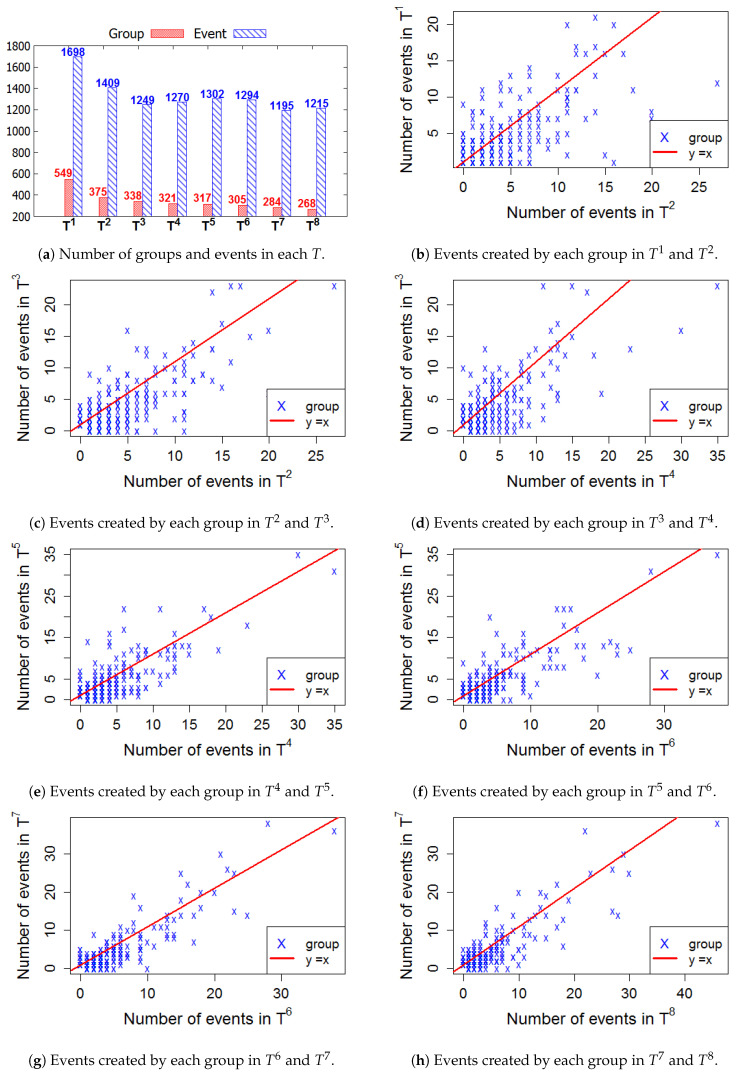
San Francisco—The numbers of groups and events in 8 time windows.

**Figure 8:**
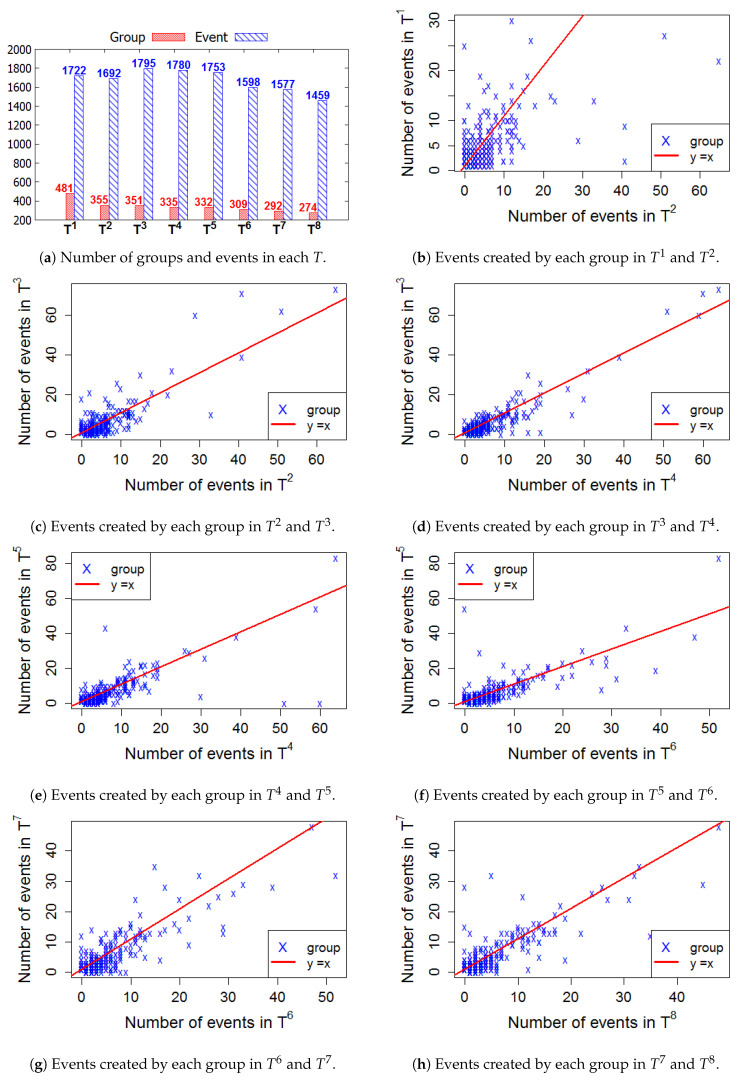
London—The numbers of groups and events in 8 time windows.

**Figure 9:**
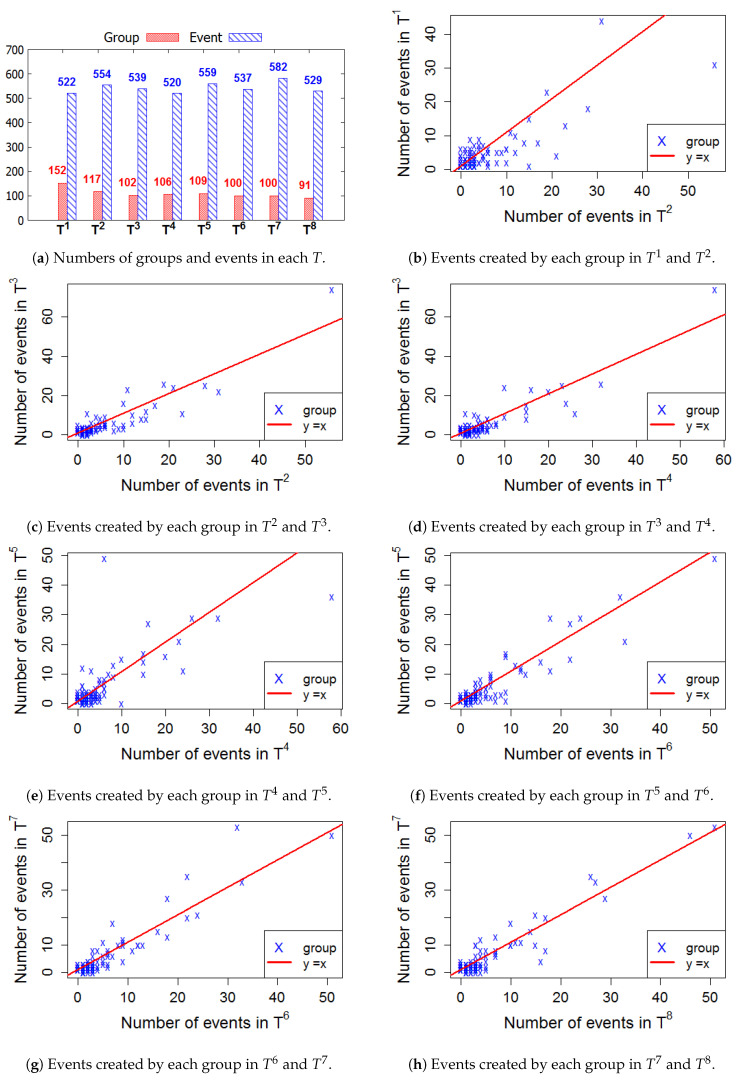
Sydney—The numbers of groups and events in 8 time windows.

Figure 6, Figure 7, Figure 8 and Figure 9 present the numbers of groups and the total numbers of events created by those groups in each $T^{i}$ of the four cities. These four figures also describe the changes in the numbers of events created by each group between two consecutive *T*. In these figures, each X denotes a group. The straight line represents the points where the numbers of events in two consecutive windows are equal. In general, the numbers of those groups steadily decrease in the 2-year lifetime for all 4 cities. However, the numbers of events that are held in each *T* fluctuate. This means, in a time period $T^{i}$, the number of events created by various groups sharply increased or decreased, as compared to $T^{i-1}$.

To evaluate the activeness of group *G* in terms of the number of events, we first collect all events of *G* in each *T*. We then use Equation (1) to compute *R*, the ratio of the numbers of events between two consecutive *T*, for example $T^{3}$ and $T^{4}$. We use Equation (2) to assign the activeness label of *G* between $T^{3}$ and $T^{4}$. Finally, the association *label matrix* is used to assign the activeness label for each group after several consecutive time windows.

Table 5 shows the activeness labels of all groups in the four cities during several consecutive time windows where the first column *Total* in Table 5a–d describes the numbers of groups that have at least one event in $T^{1}$. We can observe that the numbers of groups in the *Active* label achieved the highest value after one year in San Francisco and London, while those numbers in New York and Sydney achieved the highest value after one year and three months. After that, the numbers steadily deceased. Moreover, the numbers of groups with a *Stable* label have also shown a similar trend. Therefore, we can conclude that we can predict groups that are inactive or active in online social networks after one year.

### 5.3. Loyal Users

**Figure 10:**
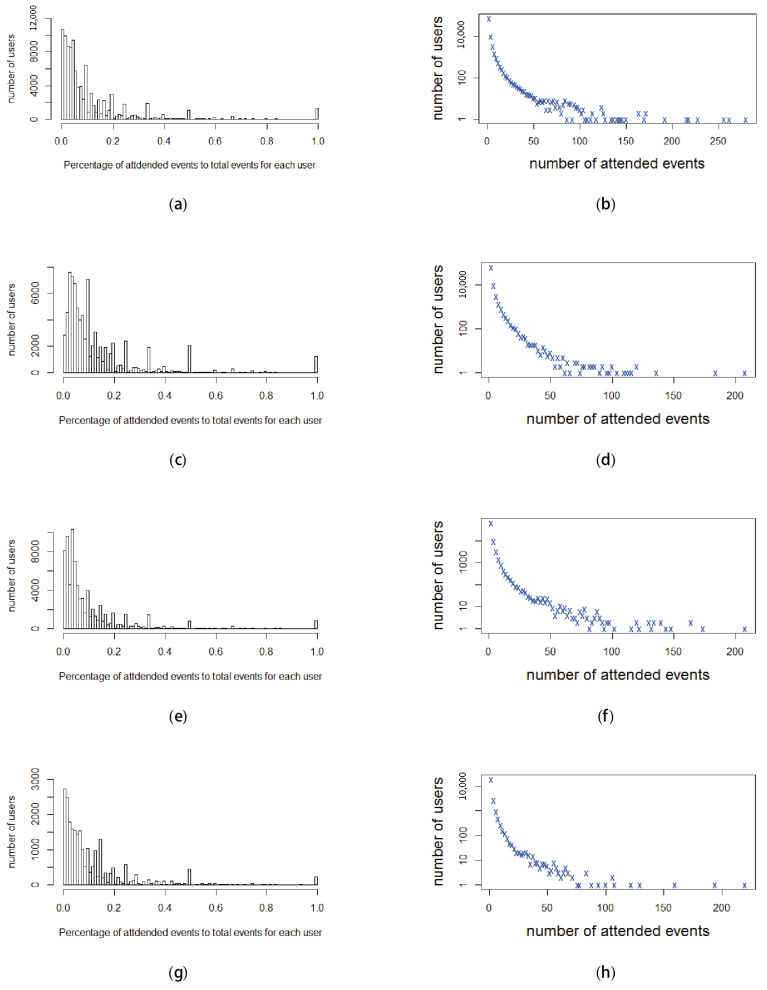
Distributions in terms of percentage and the number of attended events that users participate in among the total events of their groups in the two-year period. (**a**) New York—Percentage of attended events in total events for users. (**b**) New York—Number of attended events for users. (**c**) San Francisco—Percentage of attended events in total events for users. (**d**) San Francisco—Number of attended events for users. (**e**) London—Percentage of attended events in total events for users. (**f**) London—Number of attended events for users. (**g**) Sydney—Percentage of attended events in total events for users. (**h**) Sydney—Number of attended events for users.

Figure 10 describes the distribution of the percentage of attended events of users among the total events of their groups, and the numbers of attended events of these users in the period of two years. Specifically, Figure 10b,d,f,h demonstrates the numbers of attended events, while Figure 10a,c,e,g illustrates the percentage of the attended events of these users among the total events of their groups. We observe that the majority of users attended fewer than 10 events, as shown in Figure 10b,d,f,h. Moreover, groups of different types created various events; for example, some groups have fewer than 10 events over the two years. That is why the majority of users have a percentage of attended events among all the events of their own groups that is less than 20%, as illustrated in Figure 10a,c,e,g.

In our analysis, our aim is to clarify the relationship between the loyalty of users and the growth of their group over time, so that we evaluate the loyalty of users who attend at least one event in the first time window, $T^{1}$. If a user does not attend any event in $T^{1}$, the user will be removed in the process of evaluation. Otherwise, the user will be selected to compute the loyalty. Hence, to measure the loyalty of a user *u* in $T^{1,\cdots ,i}$, we use Equation (3) to obtain value λ. We then set threshold τ to 0.5, and the user *u* is loyal within $T^{1,\cdots ,i}$, if λ⩾τ. Otherwise, *u* is disloyal to group *G* within $T^{1,\cdots ,i}$.

**Figure 11:**
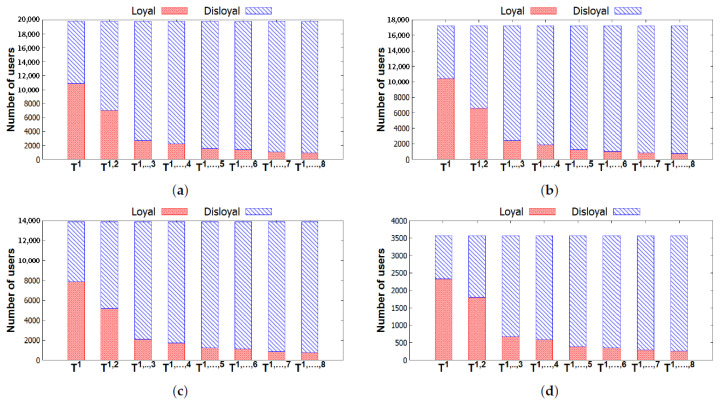
Numbers of loyal users and disloyal users varying in different Ts for the four cities. (**a**) New York—Number of loyal users and disloyal users. (**b**) San Francisco—Number of loyal users and disloyal users. (**c**) London—Number of loyal users and disloyal users. (**d**) Sydney—Number of loyal users and disloyal users.

**Figure 12:**
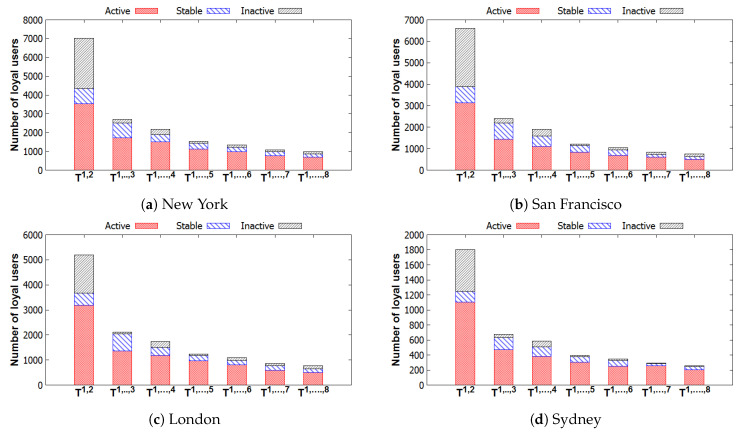
Number of loyal users in groups varying in different Ts. Groups are obtained from Table 5.

Figure 11 illustrates the number of loyal users and the number of disloyal users of all groups in each city. We can observe that the number of users who are loyal to their groups decreases by time. To understand the inactiveness of groups, we investigate the distribution of these loyal users in the groups, which is shown in Table 5. Figure 12 describes the distributions of these loyal users in different groups with three activeness labels, i.e., *Inactive*, *Stable*, and *Active*. In general, the numbers of loyal users in groups with Active and those with Stable also decrease. We can observe that a majority of these loyal users attended few events or even left their groups after the first two time windows $T^{1,2}$. Thus, those users leave their group by time due to a loss of interest in their group. Furthermore, in [26], it is argued that few interesting people and low-quality content in the online groups are the main reasons for leaving. Finding people who have the same interests and sharing many things in online groups are very difficult. These are the reasons why few people stay in groups for very long. From our results, we can conclude that a high performance of recommendation systems can be achieved if only a few items are recommended in the systems [7,8,32] within a short time period.

However, there are still some groups that continue to grow. This depends on the group’s topic, as well as the members’ expansion and relationship, which we will study it in the next article, *A New Research Problem of Diffusion Growth in Event-Based Social Networks*.

### 5.4. Activeness Prediction

We use three well-known classification methods, i.e., RF, decision tree (C5.0), and SVM, to evaluate the results in Table 5. We test these groups in three different series of consecutive time windows, called three stages: $T^{1,2}$, $T^{1,\cdots ,4}$ and $T^{1,\cdots ,8}$. For each stage in each city, we have a corresponding dataset used in the experiment. Particularly, the dataset of stage $T^{1,2}$ in New York is formed by a set of generated features and activeness labels of groups obtained only in $T^{1,2}$. In the evaluation of this stage, each corresponding dataset was divided randomly into two parts: 70% for training and 30% for testing. Each classification method of RF, C5.0, and SVM was used on each dataset 100 times to produce prediction results. The accuracy of each method is obtained by averaging all results of 100 times.

Classical methods, such as Wrapper [16] and Filter [33], are often used to compute the score and remove weak features. However, these methods are not effective for high-dimensional data. Thus, we adopt a method [29] that selects a group of 10 features from high dimensional data to result in better accuracy. These feature are listed as *N_EVENTS*, *LOYAL USERS*, *RSVPs*, *AVERAGE_Y_RSVPs*, *SD_Y_RSVPs*, *AVERAGE_DAY*, *SD_DAY*, *N_EVENT_ORGANIZER*, *N_ORGANIZER*, and *N_ATTENDEES*.

Table 6 shows the classification results of groups in three activeness classes: active, stable, and inactive. In this table, Column *ALL* means all features are used in three classifiers for each stage. Column *Selected* consists of 10 selected features that we used to obtain better results compared with those of *ALL*. We can see that the results predicted by RF are the best, while C5.0 is the worst for both *ALL* and *Selected*. Furthermore, the results in *Selected* are much higher than those in *ALL*. This indicates that the group of selected features is suitable for all classifiers, and they are strong and highly correlated.

## 6. Conclusions

In this paper, we studied the activeness of groups and the loyalty of users throughout event-based social networks. For this purpose, we used a sliding time window to represent the activity of groups as well as users’ participation in each time window. To measure the activeness of groups in several consecutive time windows, we computed the ratio of the numbers of events between two consecutive time windows. We developed an association matrix to assign a suitable activeness label to the group. The loyalty of users was manifested by consistent participation in each time window. To evaluate group activeness labels in a series of consecutive time windows, we used the crawled data of that time to generate features. We then used three well-known classifiers to validate the labels. The study shows that groups that are stable and have thrived have more loyal users. In addition to this, the study also helps to predict how long the loyal users will stay.

## Figures and Tables

**Table 1:** Dataset statistics.

City	#Groups	#Events	#Users	#YES	#NO
New York	1269	28,355	591,580	331,436	105,433
San Francisco	867	14,205	342,662	245,767	66,611
London	985	17,309	610,189	246,413	108,070
Sydney	297	5980	179,081	82,399	30,075

**Table 2:** Group categories.

Alternative Lifestyle	Book Clubs	Career/Business
Cars/Motorcycles	Community/Environment	Dancing
Education/Learning	Fashion/Beatuy	Fine Arts/Culture
Fitness	Food/Drink	Games
Health/Wellbeing	Hobbies	Language/Ethnic Identity
Lgbt	Movement/Politics	Movies/Films
Music	New Age/Spirituality	Outdoors/Adventure
Paranormal	Parents/Family	Pets/Animals
Photography	Religion/Beliefs	Sci-Fi/Fantasy
Singles	Socializing	Sports/Recreation
Support	Tech	Writing

**Table 3:** The features derived from datasets.

Category	Feature	Description	Type
Group-based	CATEGORY	Corresponding category value	Integer
	N_TOPICS	Number of topics in a group	Integer
	N_USERS	Number of users in a group	Integer
	RATING	Score average of group reviews	Double
	YEAR	The year a group is created in	Integer
	MONTH	The month a group is created in	Integer
	DAY_OF_MONTH	The day a group is created on	Integer
	WEEKDAY	The weekday a group is created on	Integer
Event-based	N_EVENTS	Number of events	Integer
	RSVPs	Number of all RSVPS	Integer
	Y_RSVPs	Number of all RSVPS only with YES	Integer
	N_RSVPs	Number of all RSVPS only with NO	Integer
	AVERAGE_RSVPs	Average of all RSVPSs	Double
	SD_RSVPs	Standard deviation of all RSVPs	Integer
	AVERAGE_Y_RSVPs	Average of RSVPS only with YES	Integer
	SD_Y_RSVPs	Standard deviation of all RSVPS only with YES	Integer
	AVERAGE_N_RSVPs	Average of RSVPS only with NO	Integer
	SD_N_RSVPs	Standard deviation of all RSVPS only with NO	Integer
	AVERAGE_DAY	Average days between two consecutive events	Double
	SD_DAY	Standard deviation of numbers of days between two consecutive events	Double
	N_EVENT_ORGANIZER	Number of events that has organizers	Double
User-based	N_ORGANIZER	Number of organizers in the group	Integer
	N_ATTENDEES	Number of users who confirm at least one YES	Integer
	BIO	Number of users who have a biography	Integer
	ADDRESS	Number of users who have address information	Integer

**Table 4:** Notations.

*G*	Group G	
$T^{i}$	The *i*th time window of group *G*
*E*	Event	
$N^{i}$	The number of events created by group *G* in $T^{i}$	Integer
*R*	Ratio of $N^{i}$ and $N^{i-1}$	Double
λ	The measure of the loyalty of a user	Double

**Table 5:** The distribution of numbers of groups in three different activeness labels after several time windows in the four cities.

	Total	Inactive	Stable	Active		Total	Inactive	Stable	Active
$T^{1}$	715				$T^{1}$	549			
$T^{1,2}$	715	286	134	295	$T^{1,2}$	549	217	104	228
$T^{1,\cdots ,3}$	715	227	201	287	$T^{1,\cdots ,3}$	549	177	157	215
$T^{1,\cdots ,4}$	715	249	136	330	$T^{1,\cdots ,4}$	549	198	107	244
$T^{1,\cdots ,5}$	715	233	145	337	$T^{1,\cdots ,5}$	549	194	124	231
$T^{1,\cdots ,6}$	715	256	124	335	$T^{1,\cdots ,6}$	549	211	102	236
$T^{1,\cdots ,7}$	715	273	128	314	$T^{1,\cdots ,7}$	549	230	95	224
$T^{1,\cdots ,8}$	715	283	131	301	$T^{1,\cdots ,8}$	549	251	88	210
(**a**) New York					(**b**) San Francisco				
	**Total**	**Inactive**	**Stable**	**Active**		**Total**	**Inactive**	**Stable**	**Active**
$T^{1}$	481				$T^{1}$	152			
$T^{1,2}$	481	157	79	245	$T^{1,2}$	152	50	26	76
$T^{1,\cdots ,3}$	481	100	155	226	$T^{1,\cdots ,3}$	152	37	40	75
$T^{1,\cdots ,4}$	481	128	88	265	$T^{1,\cdots ,4}$	152	42	30	80
$T^{1,\cdots ,5}$	481	124	92	265	$T^{1,\cdots ,5}$	152	39	28	85
$T^{1,\cdots ,6}$	481	146	80	255	$T^{1,\cdots ,6}$	152	46	21	85
$T^{1,\cdots ,7}$	481	155	89	237	$T^{1,\cdots ,7}$	152	43	27	82
$T^{1,\cdots ,8}$	481	179	82	220	$T^{1,\cdots ,8}$	152	51	26	75
(**c**) London					(**d**) Sydney				

**Table 6:** Average accuracy of three methods for both *ALL* and *Selected* features generated from the three stages.

		ALL			Selected		
		**RF**	**C50**	**SVM**	**RF**	**C50**	**SVM**
	$T^{1,2}$	69.92	65.64	69.91	71.99	68.25	71.32
**NY**	$T^{1,\cdots ,4}$	74.68	71.37	74.73	77.74	75.04	76.02
	$T^{1,\cdots ,8}$	70.64	66.19	70.21	73.07	71.37	71.58
	$T^{1,2}$	69.21	66.97	69.28	72.46	69.16	70.82
**SF**	$T^{1,\cdots ,4}$	71.13	68.37	72.21	75.63	72.57	74.21
	$T^{1,\cdots ,8}$	71.47	67.69	69.72	74.59	72.94	72.15
	$T^{1,2}$	69.22	61.72	71.58	70.58	65.29	73.02
**LD**	$T^{1,\cdots ,4}$	71.5	67.61	72.15	74.66	72.51	73.82
	$T^{1,\cdots ,8}$	69.15	62.07	68.73	70.99	67.88	69.85
	$T^{1,2}$	66.1	60.34	64.71	66.33	65	68.21
**SN**	$T^{1,\cdots ,4}$	73.04	68.8	71.41	74.84	71.52	75.15
	$T^{1,\cdots ,8}$	69.71	60.86	66.31	68.32	64.73	71.54
